# Bedtime Stress Increases Sleep Latency and Impairs Next-Day Prospective Memory Performance

**DOI:** 10.3389/fnins.2020.00756

**Published:** 2020-07-28

**Authors:** Zoë-lee Goldberg, Kevin G. F. Thomas, Gosia Lipinska

**Affiliations:** ACSENT Laboratory, Department of Psychology, University of Cape Town, Cape Town, South Africa

**Keywords:** prospective memory, stress, sleep, cortisol, intention implementation

## Abstract

The cognitive construct of prospective memory (PM) refers to the capacity to encode, retain and execute delayed intentions (e.g. to remember to buy milk on the way home). Although previous research suggests that PM performance is enhanced by healthy sleep, conclusions tend to be drawn based on designs featuring ecologically unnatural manipulations (e.g. total sleep deprivation). This study investigates whether a more common everyday experience (bedtime stress) affects next-day PM performance and, in so doing, also contributes to the heretofore inconsistent literature on stress and PM. Forty young adults received PM task instructions and were then assigned to either a stress condition (exposure to a laboratory-based stress-induction manipulation; *n* = 20, 9 women) or a non-stress condition (exposure to a non-stressful control manipulation; *n* = 20, 12 women). After completing the experimental manipulation, all participants had their objective sleep quality measured over a full night of polysomnographic monitoring. Upon awakening, they completed the PM task. Analyses detected significant between-group differences in terms of stress outcomes, sleep quality and PM performance: Participants exposed to the manipulation experienced heightened signs of stress (captured using a composite variable that included self-report, psychophysiological and endocrinological measures), had longer sleep latencies and poorer sleep depth and displayed significantly longer reaction times to PM cues. An interaction between experimental condition (being exposed to the stressor) and disrupted sleep (longer sleep latency) significantly predicted poorer next-day PM reaction time. We interpret these findings as indicating that bedtime stress, which leads to heightened presleep arousal, affects sleep processes and, consequently, the deployment of attentional resources during next-day execution of a delayed intention.

## Introduction

Prospective memory (PM) is a cognitive construct describing processes involved in the formation, retention and execution of delayed intentions. In other words, it is memory for future goal-directed behaviour (McDaniel and Einstein, 2007). Successful PM performance requires a specific intention to be encoded and stored so that the likelihood of retrieval is heightened upon detection of an environmental cue (e.g. a particular event, place, activity or time; Kliegel et al., 2001). This retrieval can be spontaneous (i.e. using reflexive–associative memory processes, the cue triggers automatic retrieval of the intention from long-term storage) or strategic (i.e. active monitoring of the environment for relevant cues, with effortful reliance on attentional resources; McDaniel and Einstein, 2000).

Healthy sleep enhances PM performance because spontaneous retrieval processes are especially likely to benefit from sleep-dependent consolidation of the encoded intention (Scullin and McDaniel, 2010; Diekelmann et al., 2013b; Barner et al., 2017). PM performance is poorer and relies more heavily on strategic monitoring when a period of total sleep deprivation separates intention encoding from retrieval and execution (Grundgeiger et al., 2014; Esposito et al., 2015; Occhionero et al., 2017). Moreover, specific sleep stages might differentially support consolidation of the encoded PM intention. Diekelmann et al. (2013a) showed that participants who experienced an interval of early-night slow-wave (SWS)-rich sleep between encoding and retrieval/execution were more likely to respond accurately to PM cues than those who experienced late-night rapid eye movement (REM)-rich sleep during that interval.

Hence, a growing body of evidence supports the conclusion that healthy sleep (and particularly, perhaps, uninterrupted early-night sleep) benefits PM performance. This evidence is, however, based on experimental manipulations (total sleep deprivation, or exclusive experience of one type of sleep stage) that do not necessarily mirror natural ecological conditions. Here, we turn the focus to ways in which a more common everyday experience (viz. bedtime stress) might affect PM performance.

Studies examining effects of laboratory-induced stress on PM performance have delivered inconsistent results. For instance, whereas some (Nater et al., 2006; Walser et al., 2013; Möschl et al., 2017) report that stress exposure has no effect on event-based PM, others (Glienke and Piefke, 2016; Szöllösi et al., 2018) report enhanced poststress performance on such tasks. However, none of those study designs featured a significant delay between intention encoding and retrieval/execution, and in most cases, both phases of the PM process were instantiated under stressful conditions.

Although a relatively large literature examines relations between the experience of chronic daytime stress and sleep disruption (Hall et al., 2007; Mezick et al., 2009; Hall et al., 2017), few studies have examined whether cognitive processes are affected by the experience of laboratory-induced stress immediately before bedtime (and none have examined whether next-day retrieval/execution of a PM intention encoded prior to sleep is affected by such an experience). This question is of interest because self-reported bedtime stress affects sleep quality negatively (Åkerstedt et al., 2012), and laboratory-induced acute psychosocial stress experienced immediately prior to a nap increases sleep latency and decreases slow-wave activity (Ackermann et al., 2019). The mechanisms underlying these effects appear to be related to the increases in sympathetic arousal and cortisol (CORT) concentrations provoked by the experience of stress (McEwen et al., 2015; Sapolsky, 2015). These elevated CORT concentrations are of particular relevance here because (a) when night-time CORT concentrations are artificially increased, there tend to be specific effects on sleep stages (viz. SWS) during which the lowest diurnal levels of that hormone are typically observed and during which processes critical to memory consolidation typically occur (Wagner et al., 2005; Henry et al., 2018) and (b) brain regions with dense assemblages of glucocorticoid receptors (e.g. prefrontal cortex; de Kloet et al., 2018) are heavily involved in PM processes (Cona et al., 2015).

The present study therefore investigated whether exposure to a laboratory-based stress-induction manipulation immediately after encoding a PM intention and immediately prior to bedtime would disrupt sleep quality and affect next-morning PM performance negatively. We tested these specific hypotheses: (1) participants exposed to the stressor will experience a physiological stress response and report increased subjective stress postexposure, display disrupted patterns of sleep architecture and demonstrate relatively poor PM task performance and (2) an interaction between stress exposure and sleep disruption will account for a significant portion of the variance in PM performance.

## Methods

### Participants

We recruited undergraduate students from a departmental subject pool. Seventy volunteers, all of whom received course credit, participated in the initial screening. Twenty-five did not meet eligibility criteria [i.e. prior or current diagnosis of any major mental or neurological condition likely to affect cognition, as characterised by the Diagnostic and Statistical Manual of Mental Disorders 5th Edition (DSM-5) or use of sleep regulatory or psychoactive medications] to progress to the sleep study and five more withdrew postscreening. The remainder (*N* = 40) were assigned into either a non-stress (8 men, 12 women) or stress group (11 men, 9 women), with each male and female alternately allocated to ensure that the gender distribution was relatively equal across groups.

## Materials and Procedure

Study procedures were conducted in a university sleep laboratory. Each participant was run individually, with a single screening session preceding the main procedures by approximately 3 weeks. Ethical approval was granted by our institution’s Research Ethics Committee, and all protocols adhered to Declaration of Helsinki (World Medical Association, 2013) guidelines.

### Preliminary Clinical Screening

The Mini International Neuropsychiatric Interview version 7.0.2 (Sheehan et al., 1998) assessed whether potential participants were free of major psychiatric and substance use disorders. For corroboration, we administered the Beck Depression Inventory, Second Edition (BDI-II; Beck et al., 1996), excluding those scoring > 14; the Michigan Alcoholism Screening Test (Shields et al., 2007), excluding those scoring > 5 and the Drug Abuse Screening Test (Skinner, 1982), excluding those scoring > 5. The Pittsburgh Sleep Quality Index (PSQI; Buysse et al., 1989) characterised subjective sleep quality.

### Main Study Procedures

**FIGURE 1:**
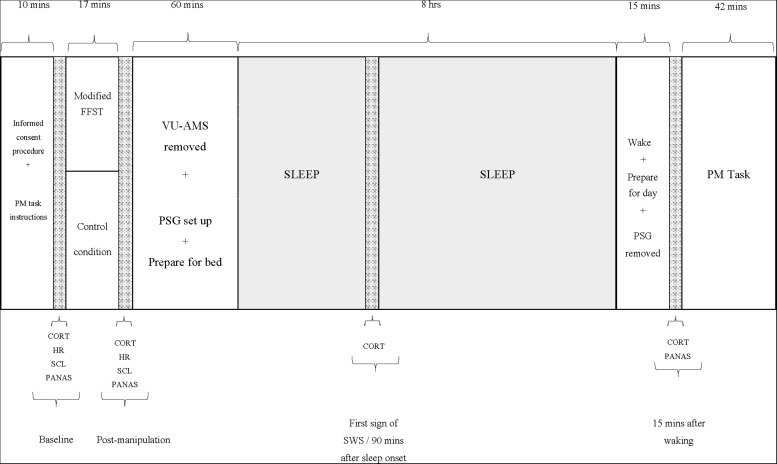
Study procedure. PM, prospective memory; FFST, Fear Factor Stress Test; VU-AMS, Vrije Universiteit Ambulatory Monitoring System; PSG, polysomnograph; CORT, salivary cortisol measure; HR, heart rate measure (an average over 5 min); SCL, skin conductance measure (an average over 5 min); PANAS, positive and negative affect scale; SWS, slow-wave sleep.

Figure 1 depicts these procedures.

The participant arrived at the laboratory between 18h30 and 19h30. Individual participants were scheduled in a way that allowed for them to be in bed and awakened within 30 min of their habitual bed and waketimes. A researcher assisted in completion of informed consent procedures and orientation to the sleep laboratory environment. The participant then received instructions for the PM task and completed the Trait form of the State-Trait Anxiety Inventory (STAI; Spielberger et al., 1983). Thereafter, the researcher connected the participant to the Vrije University Ambulatory Monitoring System (VU-AMS; Klaver et al., 1994) and took baseline (i.e. average across the first 5 min after connection) measures of heart rate (HR) and skin conductance level (SCL). The participant then completed the items measuring negative mood states from the Positive and Negative Affect Schedule (PANAS; Watson et al., 1988) to characterise the extent to which they were experiencing unpleasant and distressing emotions at baseline and followed that by chewing on a Salivette^®^ swab to provide the first saliva sample from which CORT concentrations would be assayed.

Participants then entered the manipulation phase. Those assigned to the stress group completed a slightly modified version of the Fear Factor Stress Test (FFST; du Plooy et al., 2014). This laboratory-based psychosocial/physiological stressor comprises four components: (a) a 5-min speech preparation period that encompasses a mortality salience (MS) manipulation (i.e. the participant is asked to write a description of their own death, with the instruction that this writing will subsequently be delivered verbally to an audience of judges); (b) a 5-min speech delivery period that required the participant to perform the prepared speech without prepared notes); (c) a 5-min mental arithmetic task that required consecutive subtractions of 17, starting at 2043 and (d) a 2-min physical task that required submersion of the dominant hand in a bucket of ice water (between 0 and 4°C) for as long as possible, up to 2 min (Adams and Minnozzi, 2013). The essential difference between this stress manipulation and the FFST is the MS component. Previous social psychological research has investigated the effects of MS manipulations on a range of human behavior (see, e.g. Klackl and Jonas, 2019), and at the neurobiological level, there is evidence that MS induces an orienting response that provokes a cascade of neurobiological events, including the release of CORT (Tritt et al., 2012).

Those assigned to the non-stress group completed an equivalent control condition. They were asked to (a) take up to 10 min to write a summary of their day’s activities and to then read this summary to the examiner; (b) complete a simple 5-min mental arithmetic task (consecutive additions of 5, starting at 0) and (c) place the dominant hand into a bucket of warm water (34–38°C) for up to 2 min.

After the conclusion of the experimental manipulation, the participant provided a second saliva sample, and the researcher took a second set of HR and SCL measures (again, an average over 5 min). The researcher then disconnected the VU-AMS, and the participant then completed the PANAS for a second time. Thereafter, the researcher prepared the participant for an 8-h period of polysomnographically (PSG) monitored sleep. To ensure the integrity of all records, we implemented a bipolar longitudinal montage, including the bipolar derivations F3-C3, C3-P3, P3-O1 and F4-C4, C4-P4, P4-O2 in combination with a referential montage using F3-A2, C3-A2, O1-A2 and F4-A1, C4-A1, O2-A1 derivations. When the participant entered the first phase of SWS or after 90 min of sleep (depending on which came first), the researcher woke the participant briefly and collected the third saliva sample. This sampling method has been used previously in prior research (Henry et al., 2018).

Approximately 15–20 min after waking, the researcher collected the final saliva sample. Immediately thereafter, the participant completed the PANAS for a final time, and the researcher removed the PSG equipment.

The participant was then administered the PM task, which was embedded within a computer-based general knowledge questionnaire (McFarland et al., 2016). A conventional computer monitor presented 196 general knowledge questions, each featuring four response options (A, B, C or D). Participants were instructed to select the correct answer by pressing the appropriate key. Eight target PM trials, each signalled by the word ‘president’, were included within the 196 questions. When this word appeared, participants were required to press the number 6 key rather than one of the letter keys. The software recorded PM-trial responses and their corresponding reaction times.

After completing the PM task, participants were debriefed and remunerated approximately $14. Participants were not allowed to consume any caffeinated food or drinks during the study protocol.

### Statistical Analyses

We analysed data using SPSS (version 25.0), with the threshold for statistical significance at α = 0.05. Outliers within each dataset were removed according to Tukey (1977) criteria.

Inferential analyses proceeded across six steps. First, a series of independent-sample *t*-tests characterised between-group differences in sociodemographic and clinical characteristics. Second, a 2 (group: stress, non-stress) × 2 (sex: male, female) factorial ANOVA investigated whether the modified FFST was successful in inducing a stress response. We included biological sex as a factor here because previous research suggests that there are sex differences in laboratory-induced physiological stress responses (Reschke-Hernández et al., 2017). To decrease the probability of producing a type I error, and to account for the relative lack of statistical power due to the small sample size, we created a composite outcome variable for this analysis that captured both subjective and objective stress measures. To calculate this stress composite, we subtracted baseline PANAS, HR, SCL and CORT values from their respective postmanipulation values, standardised each of those variables and took the average across the *z*-scores. Third, a series of independent-sample *t*-tests investigated between-group differences in persistence of the stress response across the night. Here, the three outcome variables were (a) PANAS morning score minus PANAS at baseline, (b) CORT at the SWS measure minus CORT at baseline and (c) CORT at the morning measure minus CORT at baseline. Fourth, another series of independent-sample *t*-tests characterised between-group differences on the following set of PSG-measured sleep outcome variables: sleep latency, sleep efficiency, percentage of non-rapid eye movement (NREM) sleep stages 1–3 [NREM1 percentage, NREM2 percentage, NREM3 (SWS) percentage], REM latency, REM percentage, awakenings after sleep onset (WASO), microarousals and sleep depth {estimated by *M*[*z*(NREM1%) + *z*(SWS%)]}; Baglioni et al., 2016. To obtain the values supporting these variables, we classified sleep stages according to the American Academy of Sleep Medicine (Berry et al., 2017) guidelines. Fifth, independent-samples *t*-tests characterised between-group differences in PM accuracy (a raw score out of 8) and average PM reaction time (RT) across the eight trials. Sixth, two separate general linear models (GLMs) investigated whether group membership, stress (as indexed by the stress composite variable), sleep quality and/or two- and three-way interactions significantly predicted PM accuracy and RT, respectively. To decrease the probability of producing a type I error, and to account for the relative lack of statistical power due to the small sample size, we included in the model-building process only those sleep outcome variables for which previous analyses had detected significant between-group differences (*p* < 0.05, one-tailed). We worked iteratively to find the best-fitting model, removing non-significant interactions first, then non-significant main effects, before evaluating the statistical significance of the overall fit. Bivariate correlations or *t*-tests explored significant main or interaction effects within that best-fitting model.

## Results

### Sample Characteristics

Analyses detected no significant between-group differences with regard to sex distribution or to STAI-Trait, BDI-II and PSQI scores (Table 1). Although, by design, all participants were aged between 18 and 25 years, on average, those in the stress group were significantly younger than those in the non-stress group. However, this between-group difference is unlikely to be ecologically relevant because the magnitude of the mean difference was only about 1.3 years.

**TABLE 1:** Sample demographic and clinical characteristics (*N* = 40).

	**Experimental condition**					
	**Stress**	**Non-stress**				
**Measure**	**(*n* = 20)**	**(*n* = 20)**	**Range**	***t*/χ^2^**	***p***	**ESE**
Age^a^	19.06 (1.11)	20.32 (2.00)	18–25	2.35	0.03*	0.77
Sex (M/F)	11:9	8:12	–	0.90	0.34	0.15
BDI-II^b^	5.61 (5.49)	5.31 (5.94)	0–12	0.15	0.88	0.05
STAI-Trait	53.25 (3.40)	52.70 (4.45)	41–62	−0.44	0.66	0.14
PSQI^b^	7.89 (3.68)	6.13 (3.05)	0–19	1.51	0.14	0.52

For continuous variables, the second and third columns present means, with standard deviations in parentheses. For the categorical variable, those columns present raw frequencies. STAI-Trait, State-Trait Anxiety Inventory Form Y-2 (total score reported); BDI-II, Beck Depression Inventory, Second Edition (total score reported); PSQI, Pittsburgh Sleep Quality Index (total score reported); ESE, effect size estimate (in this case, Cohen’s *d* for *t*-tests and Cramer’s *V* for χ^2^ tests). ^*a*^Two participants in the stress group and one participant in the non-stress group declined to report their ages; hence, data are based on *n* = 18 for the former and *n* = 19 for the latter. ^*b*^Two participants in the stress group and four participants in the non-stress group did not answer all questions on these instruments; hence, data are based on *n* = 18 for the former and *n* = 16 for the latter. *p < 0.05. All *p*-values are two-tailed.

### Manipulation Check

Table 2 presents raw data for all components of the stress composite variable and descriptive statistics for that standardised variable.

**TABLE 2:** Self-reported and physiological stress: descriptive statistics (*N* = 40).

	**Experimental condition**	
	**Stress**	**Non-stress**
**Measure**	**(*n* = 20)**	**(*n* = 20)**
PANAS—negative scale		
Baseline	13.35 (3.10)	14.05 (4.90)
Postmanipulation	16.25 (5.46)	13.00 (5.25)^a^
Morning	11.35 (2.28)	11.30 (2.16)
Heart rate^b^		
Baseline	91.10 (17.52)	79.45 (10.40)
Postmanipulation	101.47 (18.45)	77.83 (10.66)
Skin conductance level^c^		
Baseline	3.21 (2.84)	4.41 (5.40)
Postmanipulation	5.25 (4.10)	4.41 (5.66)
Cortisol^d,e^		
Baseline	3.90 (2.31)	1.71 (1.73)
Postmanipulation	5.65 (5.35)	2.26 (1.67)
Sleep	3.32 (2.65)	2.03 (2.15)
Morning	14.01 (11.02)	8.36 (5.45)
Stress composite^f^	0.46 (0.53)	−0.52 (0.22)

*Means are presented with standard deviations in parentheses. PANAS, positive and negative affect schedule. ^*a*^One participant in this group did not answer all questions on this instrument, hence *n* = 19. ^*b*^Measured in beats per minute (bpm). ^*c*^Measured in microSiemens (μS). ^*d*^Measured in nanomoles per liter (nmol/L). ^*e*^Some saliva samples were corrupted, and other values were excluded as outliers. Hence, baseline data are based on *n* = 18 for the stress group and *n* = 19 for the non-stress group; postmanipulation data are based on *n* = 19 for the stress group and *n* = 9 for the non-stress group; sleep data are based on *n* = 16 for the stress group and *n* = 18 for the non-stress group; and morning data are based on *n* = 16 for the stress group and *n* = 19 for the non-stress group. ^*f*^Due to the missing data described above, *n* = 19 for the stress group (10 men, 9 women) and *n* = 17 for the non-stress group (7 men, 10 women).*

Analyses of the stress composite data detected a significant main effect of experimental condition, *F*(1,32) = 49.10, *p* < 0.001, η_p_^2^ = 0.61, but no significant main effect of sex, *F*(1,32) = 0.40, *p* = 0.53, η_p_^2^ = 0.01, and no significant interaction effect, *F*(1,32) < 0.001, *p* = 0.99, η_p_^2^ < 0.001. This set of statistics suggests that (a) exposure to the modified FFST provoked a significant postmanipulation spike in stress responses, (b) exposure to the control condition did not do the same and (c) men and women responded similarly to the experimental condition to which they had been exposed.

The initial between-group differences in subjective and objective stress did not persist through the night. Analyses detected no significant between-group differences in baseline-controlled PANAS scores at the morning measurement point or in baseline-controlled CORT concentrations at both the SWS and morning measurement points (see Table 3). This result was expected because physiological responses to an acute stressor (even those involving the long-acting hormone CORT) tend to dissipate within 2 h of stimulus offset (Lopez-Duran et al., 2014).

**TABLE 3:** Between-group differences: persistence of the stress response across the night (*N* = 40).

	**Experimental condition**					
	**Stress**	**Non-stress**				
**Outcome variable**	**(*n* = 20)**	**(*n* = 20)**	***t***	***p***	**95% CI (LL, UL)**	**ESE**
PANAS—negative scale						
Δ(morning - baseline)	−2.00 (3.39)	−2.75 (3.68)	−0.67	0.51	−3.02, 1.52	0.21
Cortisol^a^						
Δ(sleep - baseline)^b^	0.11 (3.20)	−1.00 (1.21)	−0.23	0.81	−2.13, 1.71	0.46
Δ(morning - baseline)^c^	9.45 (8.86)	7.48 (7.83)	−0.68	0.50	−7.92, 3.97	0.24

*The second and third columns present *M*(*SD*). All *p*-values are two-tailed. PANAS, positive and negative affect schedule; CI, confidence interval; LL, lower limit; UL, upper limit; ESE, effect size estimate (in this case, Cohen’s *d*). ^*a*^Measured in nanomoles per litre (nmol/L). ^*b*^Because some saliva samples were corrupted and others were excluded as outliers, data here are based on *n* = 14 for the stress group and *n* = 16 for the non-stress group. ^*c*^Data based on *n* = 19 for the stress group and *n* = 14 for the non-stress group; reasons for the missing data are given above.*

### Sleep Architecture

**FIGURE 2:**
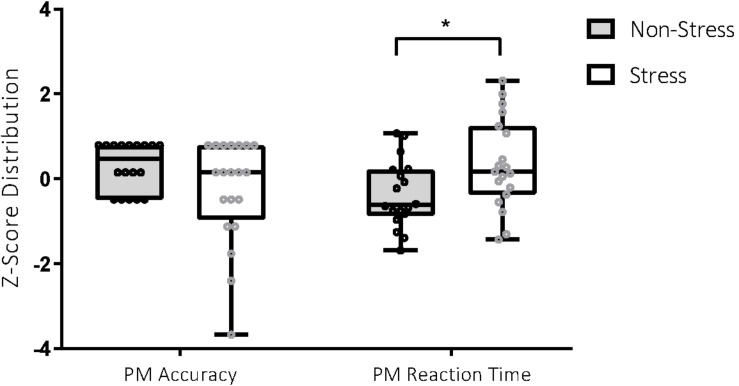
Performance on the prospective memory (PM) task in the stress and non-stress groups. For PM accuracy (score out of 8), participants in the non-stress group (*n* = 18) scored better than those in the stress group (*n* = 20), 7.22 ± 0.88 vs 6.35 ± 1.93. For PM reaction time (measured in milliseconds), participants in the non-stress group (*n* = 18) performed better than those in the stress group (*n* = 19), 4147.86 ± 1162.58 vs 5201.88 ± 1570.47. *p < 0.05.

Participants in the stress group experienced more disrupted sleep (see Figure 2). Analyses detected significant between-group differences, associated with medium-to-large effect sizes, with regard to sleep latency (those exposed to the stressor took almost twice as long to fall asleep), NREM2% and sleep depth (see Table 4).

**TABLE 4:** Between-group differences: polysomnographic measures of sleep architecture (*N* = 38).

	**Experimental condition**					
	**Stress**	**Non-stress**				
**Outcome variable**	**(*n* = 19)**	**(*n* = 19)**	***t***	***p***	**95% CI (LL, UL)**	**ESE**
Sleep latency	37.89 (28.64)^a^	21.76 (20.06)	−1.99	0.03*	−32.55, 0.30	0.66
Sleep efficiency	84.24 (9.07)	88.19 (6.95)	1.51	0.07	−1.37, 9.27	0.49
NREM1%	12.16 (5.96)	9.61 (3.63)^a^	−1.57	0.07	−5.87, 0.76	0.51
NREM2%	53.22 (5.57)^b^	45.51 (9.37)	−2.96	0.003**	−13.03, −2.42	0.99
NREM3%	22.85 (10.16)	27.82 (9.73)	1.54	0.07	−1.57, 11.52	0.50
REM latency	139.89 (72.32)	118.25 (56.76)	−1.03	0.16	−64.42, 21.13	0.33
REM%	14.30 (5.42)	15.94 (4.94)	0.97	0.17	−1.78, 5.05	0.32
WASO	41.89 (37.99)	31.29 (23.78)	−1.03	0.16	−31.46, 10.25	0.33
Microarousals	70.79 (22.83)	69.95 (21.70)	−0.12	0.46	−15.50, 13.81	0.04
Sleep depth	−0.25 (0.92)	0.31 (0.62)^a^	2.14	0.02*	0.03, 1.08	0.71

One dataset in each group was lost due to corrupted sleep data files. All data presented are percentage of total night scores, except for WASO and microarousals, which are measured in minutes. The second and third columns present *M*(*SD*). CI, confidence interval; LL, lower limit; UL, upper limit; ESE, effect size estimate (in this case, Cohen’s *d*); WASO, wake after sleep onset (i.e. the number of times the participant woke from sleep during the night). ^*a*^Data based on *n* = 18; one dataset removed because of outlying values. ^*b*^Data based on *n* = 17; two datasets removed because of outlying values. *p < 0.05. **p < 0.01. All p-values are one-tailed, given that we held a directional hypothesis (viz. sleep quality would be more disrupted in the stress group than in the non-stress group).

### PM Performance

**FIGURE 3:**
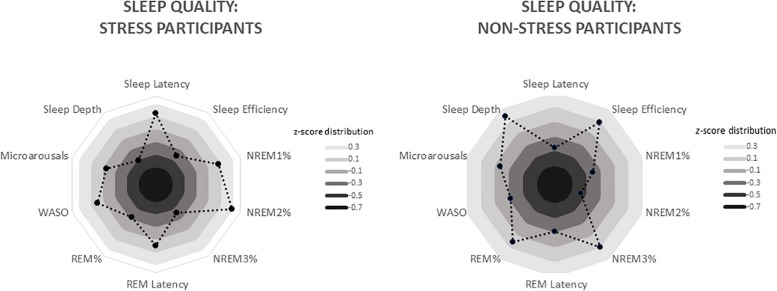
Radar charts depicting differing sleep quality in the stress and non-stress groups (*n* = 19 apiece). Data are standardised values (*z* scores), with each ring representing 0.2 *SD*. For the stress group, these values range from −0.33 (sleep depth) to 0.47 (NREM2%), whereas for the non-stress group, they range from −0.42 (NREM2%) to 0.34 (sleep depth). WASO, wake after sleep onset (i.e. number of times the participant woke from sleep during the night).

Analyses detected a significant between-group difference for PM RT, *t*(35) = −2.31, *p* = 0.03, Cohen’s *d* = 0.76. Between-group differences for PM accuracy were not statistically significant but were associated with a medium effect, *t*(36) = 1.76, *p* = 0.09, Cohen’s *d* = 0.57. Participants exposed to the stressor were less accurate and slower in their identification of PM targets, with effect sizes in the medium range (see Figure 3).

### Does Stress Exposure and/or Sleep Quality Predict PM Performance?

Regarding PM accuracy, none of the models we tested delivered a statistically significant outcome.

Regarding PM RT, the best-fitting model included the terms group and sleep latency and featured a significant two-way interaction, *F*(1,31) = 7.20, *p* = 0.012, η_p_^2^ = 0.19. Overall, the model accounted for almost 38% of the variance in the outcome, *F*(3,31) = 6.23, *p* = 0.002, *R*^2^ = 0.376. Follow-up analyses exploring the relationship between the two interacting variables found that, within the stress group, there was a significant positive correlation between sleep latency and PM RT, *r* = 0.52, *p* = 0.014 (i.e. responses to PM targets were faster with shorter time taken to fall asleep). Within the non-stress group, that relationship was in the opposite direction, although its magnitude was not statistically significant, *r* = −0.382, *p* = 0.065.

## Discussion

We investigated whether exposure to a laboratory-based stress-induction manipulation immediately after encoding an action intention and immediately prior to bedtime would disrupt sleep quality and impair next-morning retrieval and execution of that intention. Compared to their non-exposed counterparts, stress-exposed participants displayed significantly greater objective and subjective signs of stress, experienced significantly more sleep disruptions and performed significantly more poorly on the PM task. Our primary analyses then indicated that stress-exposed participants who took longer to fall asleep were most likely to be impaired on the PM task (especially in terms of reaction time).

The first step in our investigation of whether bedtime stress affects next-day PM performance via sleep-related mechanisms was to gather evidence showing that such stress affects sleep quality directly. Consistent with previous research (Ackermann et al., 2019), we found that stress-exposed participants had longer sleep onset latencies than controls. This statistically significant between-group difference is likely explained by the fact that stress group participants experienced higher levels of presleep arousal (Åkerstedt et al., 2012). The wake–sleep transition involves a gradual decline in physiological, cortical and cognitive activity (Wuyts et al., 2012). Hence, individuals with relatively higher levels of such activity (e.g. stress-exposed participants) will likely take longer to fall asleep than those with relatively lower levels (e.g. unexposed participants) and will likely have more disruptions to subsequent sleep architecture (e.g. as we observed, less sleep depth and higher NREM2 percentage).

The next step in our investigation was to analyse next-morning PM task performance. Previous studies examining effects of laboratory-induced stress on similar tasks have delivered inconsistent results (Nater et al., 2006; Walser et al., 2013; Glienke and Piefke, 2016; Möschl et al., 2017; Szöllösi et al., 2018). However, our study differs from those in two important methodological aspects: First, in those studies, participants were still experiencing the stressor’s effects (including elevated CORT levels) during retrieval and execution of the PM intention. Second, those studies did not include a sleep-filled interval between encoding and retrieval. Hence, our study is the first to examine the effects of bedtime stress on sleep quality *and* subsequent effects on next-day PM performance (i.e. at a time when participants were no longer experiencing direct and acute effects of the stressor, but when they may have been experiencing disrupted sleep as a consequence of stress exposure).

In contrast to Ackermann et al. (2019), who found that postnap performance on tasks assessing emotional and working memories were unaffected after prenap stress exposure, we found that morning PM performance was relatively impaired after presleep stress exposure (i.e. stress group participants had significantly longer reaction times to target stimuli than non-exposed participants). Moreover, our primary analyses suggested that an interaction between experimental condition and sleep latency was a significant predictor of next-day PM RT: Participants who were exposed to the stressor and who experienced lengthened sleep latency (i.e. those with inefficient downregulation of physiological, cortical and cognitive activity prior to sleep) had the longest RTs. One interpretation of this finding is that stress-exposed participants with longer sleep latencies deploy attentional resources differently when executing delayed intentions: Rather than spontaneously retrieving the PM cue, they may rely more heavily on the less efficient cognitive strategy of active environmental monitoring (Scullin and McDaniel, 2010; Möschl et al., 2017). This interpretation is consistent with the observation that the strongest impairing effects were observed for RT, with accuracy being relatively unimpaired.

The fact that PM accuracy was relatively intact is also consistent with the argument that stress × sleep interaction effects on PM performance are not routed via disruption of memory consolidation processes. Additional support for this argument is that no other sleep outcome variable (not even those, such as sleep depth and NREM2 percentage, on which analyses had detected significant between-group differences) played a significant role in predicting PM performance. If performance was negatively affected by disrupted consolidation processes, we might have observed interactions of stress exposure with, for instance, SWS activity.

These findings have several practical implications. One is that individuals who must retain prospective intentions over a sleep-filled period should ensure that they minimise stress exposure or mitigate its effects (i.e. manage arousal effectively) prior to bedtime. Another is that interventions focussed on improving long-term PM retention should include presleep relaxation techniques.

## Limitations

We acknowledge that the following design limitations constrict the extent of inferences we might draw from the observed data. First, we do not know whether, or the extent to which, the current manipulations are an ecologically valid representation of bedtime stress. The data suggest, however, that we provoked a moderate level of stress (enough to disrupt sleep, but not enough to cause pronounced sleep deprivation) and that this is an adequate representation of ordinary bedtime stress. Furthermore, the researcher administering the study protocol was not blinded to group condition, and hence, we cannot be certain whether, or to what extent, biases were introduced into the assessment process.

Second, we do not know whether participants’ stress levels and/or sleep quality were affected by the twin novelties of the experimental manipulation and a PSG-monitored laboratory sleep night. To uncouple these effects, one might invite participants to an adaptation night ahead of the experimental night to familiarise participants with the general sleep laboratory environment and protocols. An additional sleep night, also ahead of the experimental night and featuring monitored sleep but no stress manipulation at all, would help ensure that any sleep effects observed on the experimental night were due to the stress manipulation itself and not randomly occurring between-group differences. Furthermore, waking the participants during a period of sleep could have implications on both sleep quality and CORT circadian rhythm. Intraindividual stress reactivity may have clouded these results further, as the stress manipulation effects may have been concealed for individuals with already elevated basal CORT levels. Future studies could investigate these intraindividual differences more closely.

Third, we did not include a time-based PM task. These are more resource demanding and require more executive control processes and hence tend to be affected more negatively by sleep disruption (Esposito et al., 2015). We also did not include different PM tasks with varying cognitive loads. Performance on PM tasks with higher cognitive load is more vulnerable to acute psychosocial stress (Möschl et al., 2017). Future research investigating bedtime stress effects on next-day PM performance might, therefore, expand on our design by using different kinds of tasks (event as well as time based, and perhaps some that include more ecologically valid situations).

## Conclusion

This study not only adds to the body of research suggesting that healthy sleep is essential for successful cognitive performance but also makes novel contributions in its application to distinct areas of research and its relative ecological validity. We studied the common experience of bedtime stress and showed that, although the stress response did not persist throughout the night, it did affect polysomnography-monitored sleep quality (e.g. it lengthened sleep latency). We also studied the equally common experience of retaining a prospective intention over a period of sleep and showed that morning execution of that intention is slowed by an interaction of stress exposure and longer sleep latency. We interpret these findings as indicating that bedtime stress leads to heightened arousal that affects sleep processes and consequently affects deployment of attentional resources (i.e. reliance on active monitoring rather than spontaneous retrieval) during next-day PM task performance. Moreover, our results suggest that effects on morning PM performance are not accounted for by changes in slow-wave activity and related disruptions to sleep-dependent memory consolidation.

## Data Availability Statement

The datasets generated for this study are available on request to the corresponding author.

## Ethics Statement

The studies involving human participants were reviewed and approved by the University of Cape Town Research Ethics Committee. The patients/participants provided their written informed consent to participate in this study.

## Author Contributions

ZG, KT and GL formulated the research question, designed the study and analysed the data. ZG collected the data and wrote the first draft of the manuscript. KT and GL contributed to rewriting and editing. All authors contributed to the article and approved the submitted version.

## Conflict of Interest

The authors declare that the research was conducted in the absence of any commercial or financial relationships that could be construed as a potential conflict of interest.
